# Curantur or Curentur

**Published:** 1884-07

**Authors:** R. E. Dudgeon

**Affiliations:** London


### CURANTUR OR CURENTUR.

R. E. DUDGEON, M. D., LONDON.

Dr. Lippe, in his article on Dr. Hughes' lecture in the May number of your periodical, says:

"Hahnemann was a ripe classical scholar, and when he wrote *curantur* he wrote down 'a law.'"

But Hahnemann *never* wrote *curantur*, but always *curentur*, when he gave the complete homœopathic formula.

If, then, *curentur* implies that the homœopathic formula is a therapeutic rule, whereas *curantur* implies that it is a law of nature, as Dr. Lippe seems to assert, then Hahnemann's use of *curentur* would show that his idea was that the formula merely expressed a therapeutic rule.

And that this was so is evident from the words he employs in the earlier editions of the *Organon* to explain the formula. Thus in the first edition he says (Introduction, page v): "In order to cure gently, quickly, and permanently, choose in every case of disease a medium which can for itself excite a malady similar (ὅμοιον ῇάϑος) to that it has to cure (similia similibus curentur!)." Those words are a paraphrase of the formula, "Let likes be treated by likes," rather than of the phrase employed by Dr. Lippe, "Likes are cured by likes," though the latter expresses the result we hope to obtain by following the rule expressed by the former. It is doubtful, however, if the words *similia similibus curantur* can be correctly translated, "Likes are cured by likes."

The only other occasion on which, as far as I am aware, Hahnemann used the complete formula, was in his letter to the French Minister of Public Instruction (see *British Journal of Homœopathy*, xxxviii, 64), and there also he uses the word *curentur*.

Dr. Lippe says: "The sum and substance of our paper is to show that Hahnemann was the founder of Homœopathy with his own formula, *similia similibus curantur;* and that Richard Hughes, with his new formula, *similia similibus curentur*, has departed from Homœopathy hopelessly."

But Dr. Lippe has *not* shown that Hahnemann ever employed *curantur*, and I defy him to prove that he ever did so. On the contrary, I have shown that Hahnemann, on every occasion on which he used the complete formula, invariably wrote *curentur;* so, if adhesion to Homœopathy consists in the use of the formula employed by Hahnemann, it is Dr. Lippe, and not Dr. Hughes, who "has departed from Homœopathy," but I will not be so uncharitable as to add "hopelessly," for I hope he will see and express the error of his ways and adopt henceforth the true and only Hahnemannic formula—*similia similibus curentur*.

